# Synthesis and crystal structure of 5,10-bis­(phenyl­sulfon­yl)tetra­hydro­dibenzo­penta­lene

**DOI:** 10.1107/S205698902500060X

**Published:** 2025-01-28

**Authors:** Toshiki Sakami, Hikaru Watanabe, Takuma Sato, Yasuhiro Okuda, Kan Wakamatsu, Haruo Akashi, Akihiro Orita

**Affiliations:** a Graduate School of Science and Engineering Okayama University of Science, 1-1 Ridai-cho Kita-ku Okayama 700-0005 Japan; b Department of Applied Chemistry Okayama University of Science, 1-1 Ridai-cho Kita-ku Okayama 700-0005 Japan; c Department of Chemistry Okayama University of Science, 1-1 Ridai-cho Kita-ku Okayama 700-0005 Japan; d Research Institute of Frontier Science and Technology Okayama University of Science, 1-1 Ridai-cho Kita-ku Okayama 700-0005 Japan; University of Hyogo, Japan

**Keywords:** crystal structure, transannulation, ethenyl sulfone, hy­dro­genation, tetra­hydro­dibenzo­penta­lene

## Abstract

5,10-Bis(phenyl­sulfon­yl)tetra­hydro­dibenzo­penta­lene, C_28_H_22_O_4_S_2_, **1**, was suc­cessfully synthesized *via* the photocatalyst-promoted hy­dro­genative transannulation of di­sulfonyl­cyclo­octa­tetra­ene, **2**, using perylene as the photocatalyst in the presence of (*i*-Pr)_2_NEt under UV-light irradiation (398 nm, 30 W). In this reaction, the cyclo­octa­tetra­ene moiety of **2** underwent hy­dro­genative transannulation, yielding **1**. Single-crystal X-ray analysis revealed that both enanti­omers of **1** are arranged alternately along the *a* axis of the unit cell. The structure features a wide V-shaped motif consisting of 6–5–5–6 fused rings, with a dihedral angle of approximately 97.2° between the planes of the terminal phenyl­ene rings. Additionally, a pair of phenyl­sulfonyl groups were observed at the *exo* positions relative to the V-shaped array.

## Abstract 2

The structure of a curved 6–5–5–6 fused-ring system, with two benzene rings attached at both termini and a pair of phenyl­sulfonyl groups bonded to the two five-membered rings, is described.

## 1. Chemical context

Acenes have garnered significant attention for their strong inter­actions with single-wall carbon nanotubes (SWCNTs), which led to the formation of acene–SWCNT com­posites. For example, ferrocenoyl-substituted acetyl­enic anthracene (Watanabe *et al.*, 2023 ▸) and anthrylene nano tweezers (Marquis *et al.*, 2009 ▸) have been utilized to fabricate anthracene derivative–SWCNT com­posites. In both cases, multi-adsorption effects on the SWCNT surface play a pivotal role; in the former, co-operative adsorption of ferrocenoyl and acetyl­enic anthrylene moieties is essential, while in the latter, dual adsorption of V-shaped anthrylenes drives com­posite formation. The nano tweezers consist of a pair of anthrylenes connected by methyl­ene hinges. Inspired by this, we envisioned the synthesis of a new class of nano tweezer, *i.e.***1** (see Scheme), featuring a pair of aromatic rings connected by a five-membered ring-fused hinge.
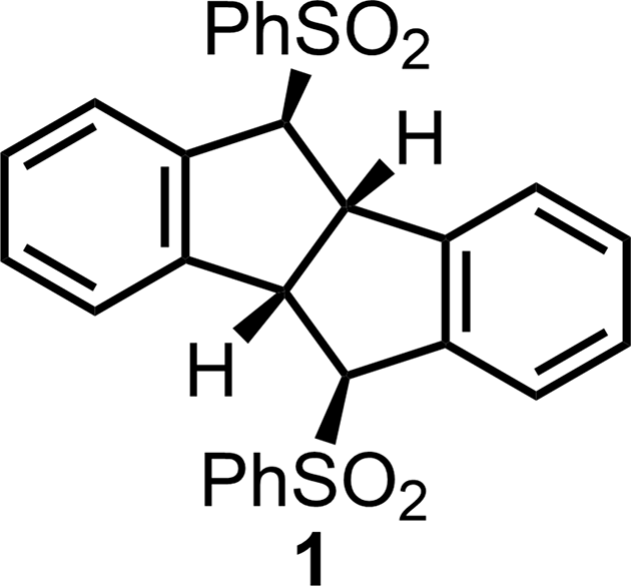


**Figure 1:**
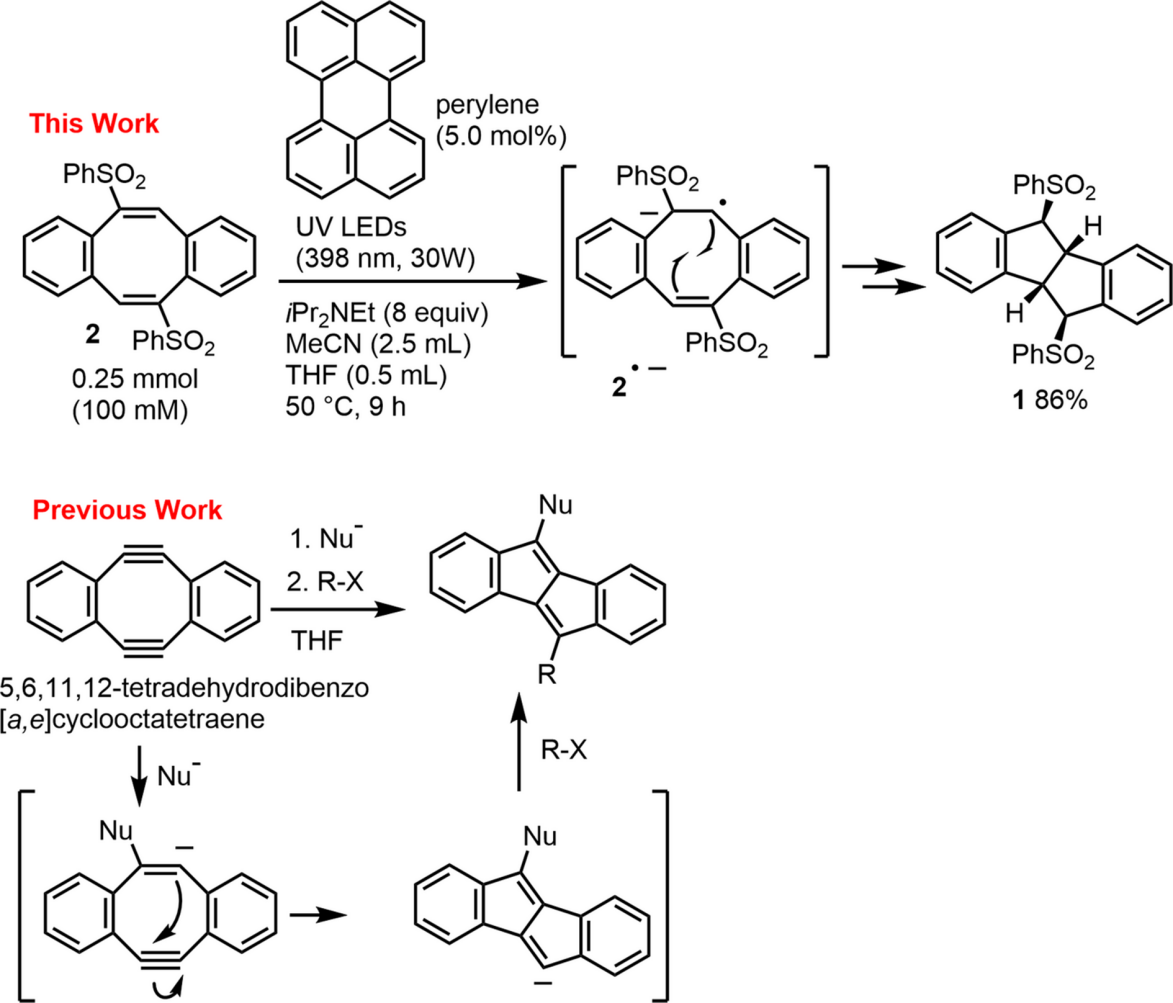
Synthetic route for the preparation of **1**.

To synthesize com­pound **1**, we employed our photocatalyst-assisted hy­dro­genative reduction protocol on 5,11-bis­(phenyl­sulfon­yl)dibenzo[*a*,*e*]cyclo­octa­tetra­ene (**2**), using a perylene photocatalyst under UV/visible-light irradiation (Watanabe *et al.*, 2020 ▸, 2021 ▸, 2024 ▸) (Fig. 1 ▸). In this reaction, we anti­cipated that the *in-situ*-formed anion radical **2**^**.**−^ would undergo transannulation to yield **1**. Notably, we have previously reported the anionic transannulation of 5,6,11,12-tetra­dehydro­dibenzo[*a*,*e*]cyclo­octa­tetra­ene, which afforded the corresponding 6–5–5–6 cyclic product dibenzo­penta­lene (Xu *et al.*, 2014 ▸). The photocatalyst-promoted hy­dro­genative transannulation of **2** proceeded successfully, yielding the nano tweezer 5,10-bis­(phenyl­sulfon­yl)tetra­hydro­dibenzo­penta­lene (**1**). In this reaction, the cyclo­octa­tetra­ene moiety of **2** was transformed into the desired five-membered ring-fused hinge.

This study presents the synthesis of 5,10-bis­(phenyl­sulfon­yl)tetra­hydro­dibenzo­penta­lene (**1**), a five-membered ring-fused nano-tweezer com­pound, along with its single-crystal X-ray structure and a plausible mechanism for the perylene/UV-light-promoted hy­dro­genative transannulation of **2**.

## 2. Structural commentary

**Figure 2:**
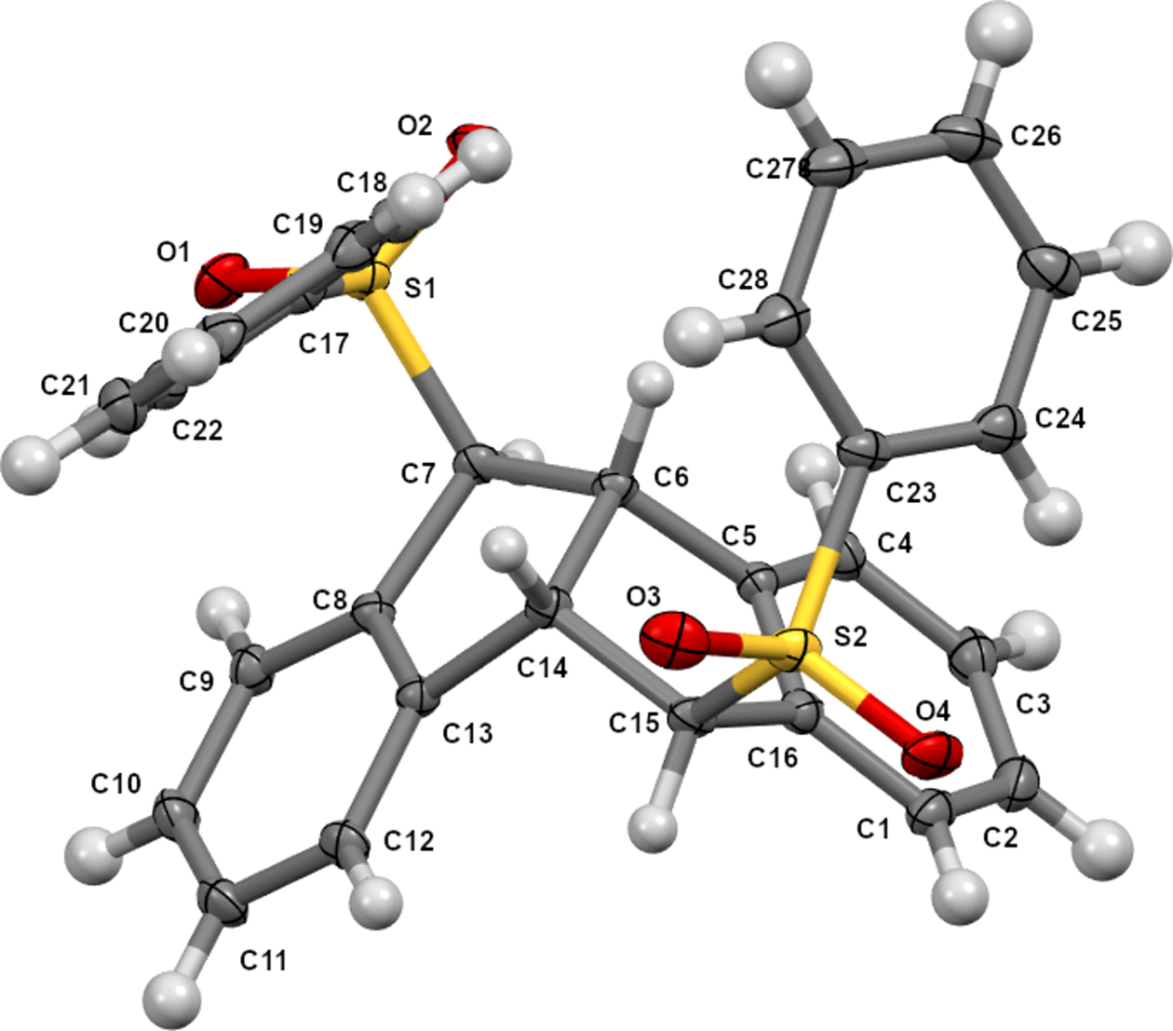
The mol­ecular structure of (*E*,*E*)-**1**, with displacement ellipsoids drawn at the 50% probability level.

The core structure of **1** is a fused 6–5–5–6-membered ring system, in which two phenyl­ene rings are connected by a five-membered-ring hinge array (Fig. 2 ▸). The dihedral angle between the planes of the terminal phenyl­ene rings is *ca* 97.2°. Phenyl­sulfonyl groups are located at the outside of the V-shaped fused-ring motif, leaning over the five-membered rings. The C1–C5/C16 (C8–C12/C13) phenyl­ene ring shows identical aromatic bond lengths (1.38–1.40 Å). In the hinge ring C6–C8/C13/C14, the C6—C7 and C6—C14 single bonds are somewhat longer than the C7—C8 and C13—C14 bonds, respectively: 1.547 (2) and 1.563 (2) Å *versus* 1.508 (3) and 1.514 (2) Å. The bond angles around the C*sp*^2^ atoms in the hinge ring [C7—C8—C13 = 110.70 (15)° and C8—C13—C14 = 111.38 (15)°] are rather larger than those around the C*sp*^3^ atoms [C6—C7—C8 = 103.59 (14)°, C7—C6—C14 = 106.00 (14)° and C6—C14—C13 = 102.05 (14)°].Similar features are observed in the other hinge ring C14–C16/C5/C6.

## 3. Supra­molecular features

**Figure 3:**
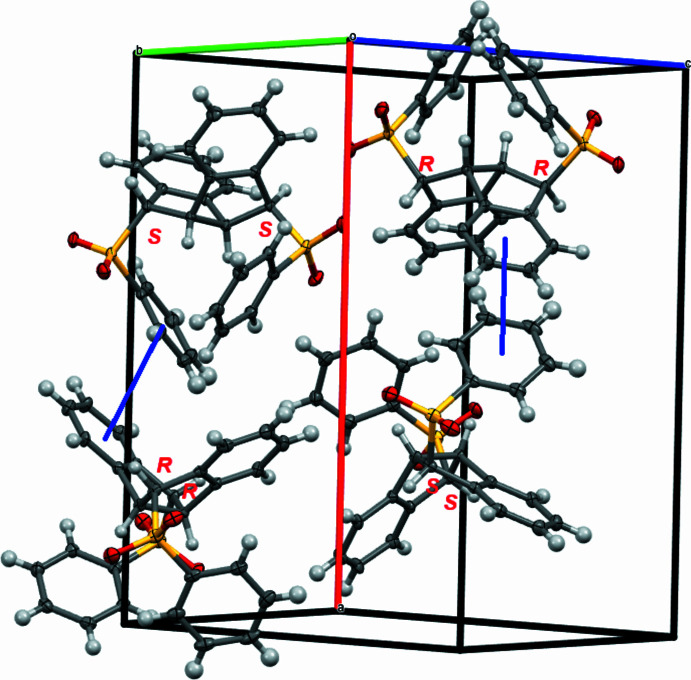
A partial packing plot of **1**, showing the linear alternating alignment of (*S*,*S*)- and (*R*,*R*)- isomers and the shortest inter­molecular contacts (blue lines).

In the crystal, **1** forms a column diagonally in the *a*-axis direction with a mol­ecular distance of 8.84 Å (Fig. 3 ▸). In the columnar structure of **1**, a pair of [(*S*)-C7, (*S*)-C15] and [(*R*)-C7, (*R*)-C15] enanti­omers are arranged alternately in the same direction, with the mid-points of the C6—C14 bonds aligned. The shortest inter­molecular contact is between the C8–C13 phenyl­ene ring and the C23′–C28′ phenyl­sulfonyl ring. The inter­molecular centroid–centroid distance between the two benzene rings is 3.86 Å, and this value is somewhat longer than conventional π–π stacking (Banerjee *et al.*, 2019 ▸).

## 4. Database survey

A search of the Cambridge Structural Database (CSD, Version 5.45, November 2023, with updates to March 2024; Groom *et al.*, 2016 ▸) indicates that 5,10-bis­(phenyl­sulfon­yl)tetra­hydro­dibenzo­penta­lene, **1**, is unprecedented. However, a related 5,10-bis­(sulfonimidoyl­meth­yl)tetra­hydro­dibenzo­pen­ta­lene derivative has been reported (CSD refcode ATUHIJ; Hermann *et al.*, 2021 ▸). The crystal structures of analogous 6–5–5–6 fused rings with carbon substituents at both the 5 and 10 positions are common, with more than 20 examples available, including the 5,10-diphenyl derivative (*e.g.* MAMYEI; Wössner *et al.*, 2022 ▸).

## 5. Synthesis and crystallization

5,10-Bis(phenyl­sulfon­yl)tetra­hydro­dibenzo­penta­lene, **1**, was successfully synthesized *via* photocatalyst perylene-promoted hy­dro­genative transannulation of di­sulfonyl­cyclo­octa­tetra­ene, **2**, in the presence of (*i*-Pr)_2_NEt under irradiation of UV light (398 nm, 30 W). Starting com­pound **2** was synthesized from the cyclic dimerization of 2-formyl­phenyl­methyl phenyl sul­fone according to the reported procedure of Xu *et al.* (2014 ▸).

To a round-bottomed flask charged with a magnetic stirrer bar were added **2** (121 mg, 0.25 mmol), perylene (3.15 mg, 12.5 µmol), (*i*-Pr)_2_NEt (0.35 ml, 2.0 mmol) and MeCN (2.5 ml). The flask was placed in a glass water bath surrounded by UV LED strip lighting, and the mixture was irradiated with UV light for 9 h. During the photoreaction, the tem­per­a­ture of the bath was kept at 50–55 °C because of heat radiation from the photoreactor. After com­pletion of the reaction, the mix­ture was evaporated and the crude product was purified by flash chromatography on silica gel (hexa­ne/EtOAc, 7:3 *v*/*v*) to afford the desired product **1** (yield: 104 mg, 0.215 mmol, 86%).

Analysis for **1**: white powder; m.p. 237–238 °C; ^1^H NMR (CDCl_3_, 400 MHz, room tem­per­a­ture): δ 3.67 (*s*, 2H), 4.62 (*s*, 2H), 7.15 (*d*, 2H, *J* = 7.8 Hz), 7.23–7.27 (*m*, 2H), 7.33 (*t*, 2H, *J* = 7.4 Hz), 7.40 (*d*, 2H, *J* = 7.8 Hz), 7.44–7.49 (*m*, 8H), 7.66–7.70 (*m*, 2H); ^13^C{^1^H} NMR (CDCl_3_, 101 MHz, room tem­per­a­ture): δ 50.4, 77.1, 124.4, 127.8, 128.5, 129.0, 129.4, 130.5, 133.5, 134.2, 136.6, 145.0. HRMS (MALDI–TOF) *m*/*z* [*M* + Na]^+^ calculated for C_28_H_22_NaO_4_S_2_ 509.0857; found 509.0807.

A crystal of **1** suitable for X-ray diffraction was obtained from the slow evaporation of an AcOEt solution.

## 6. Refinement

Crystal data, data collection and structure refinement details are summarized in Table 1 ▸. All H atoms were refined using a riding model, with *d*(C—H) = 0.93 Å, *U*_iso_(H) = 1.2*U*_eq_(C) for aromatic H, and 0.98 Å, *U*_iso_(H) = 1.2*U*_eq_(C) for CH.

## 7. Reaction mechanism

### 7.1. Mechanistic insights into hy­dro­genative transannulation *via* DFT calculations

**Figure 4:**
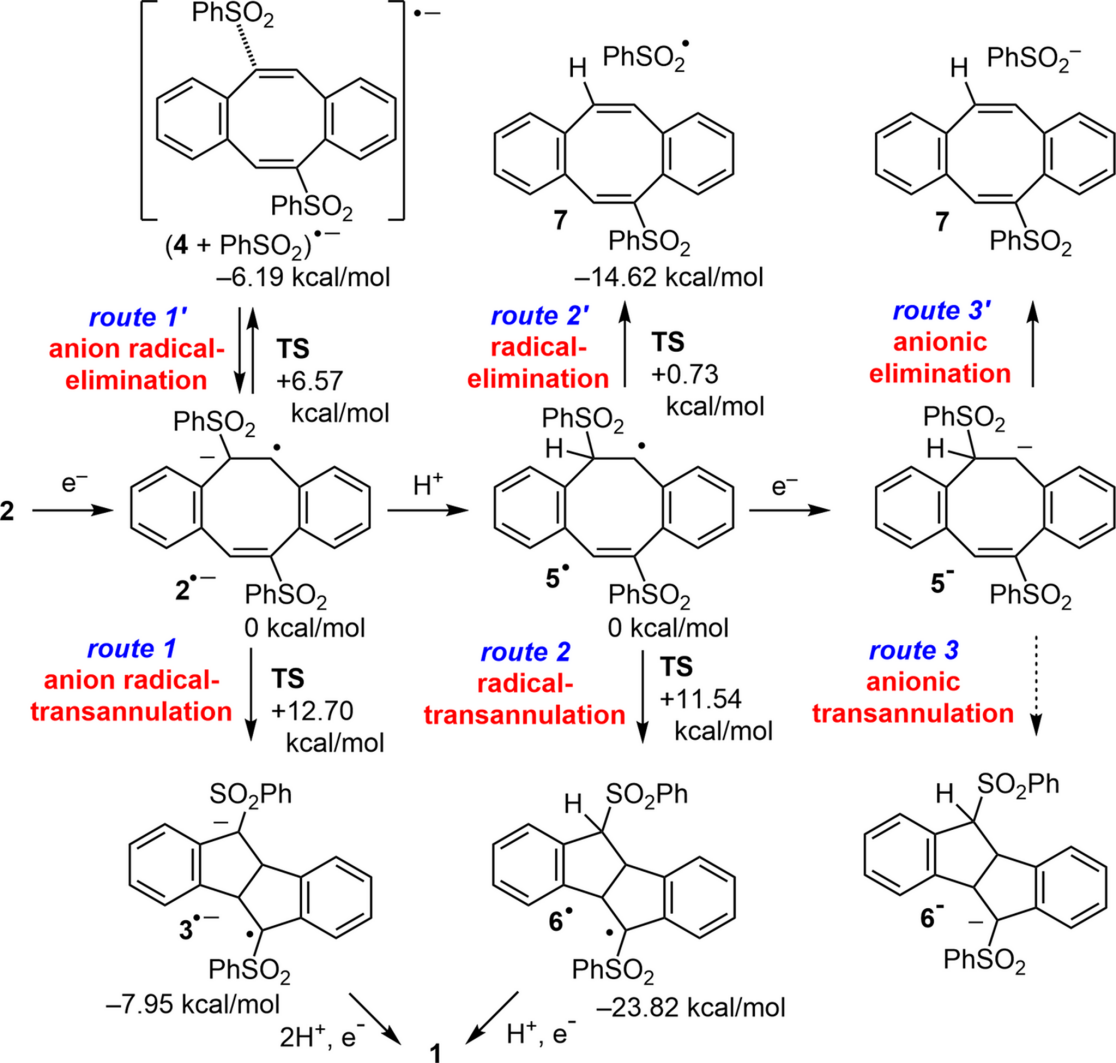
Mechanistic pathways for the transformation of **2** to **1**.

Density functional theory (DFT) calculations [B3LYP/6-31+G(d) with the IEFPCM solvent model in MeCN] were performed to elucidate the mechanism of hy­dro­genative transannulation. The results suggest that the reaction proceeds primarily *via* the anion radical **2**^**.**−^ through an anion radical-mediated mechanism (Fig. 4 ▸, route 1).

**Figure 5:**
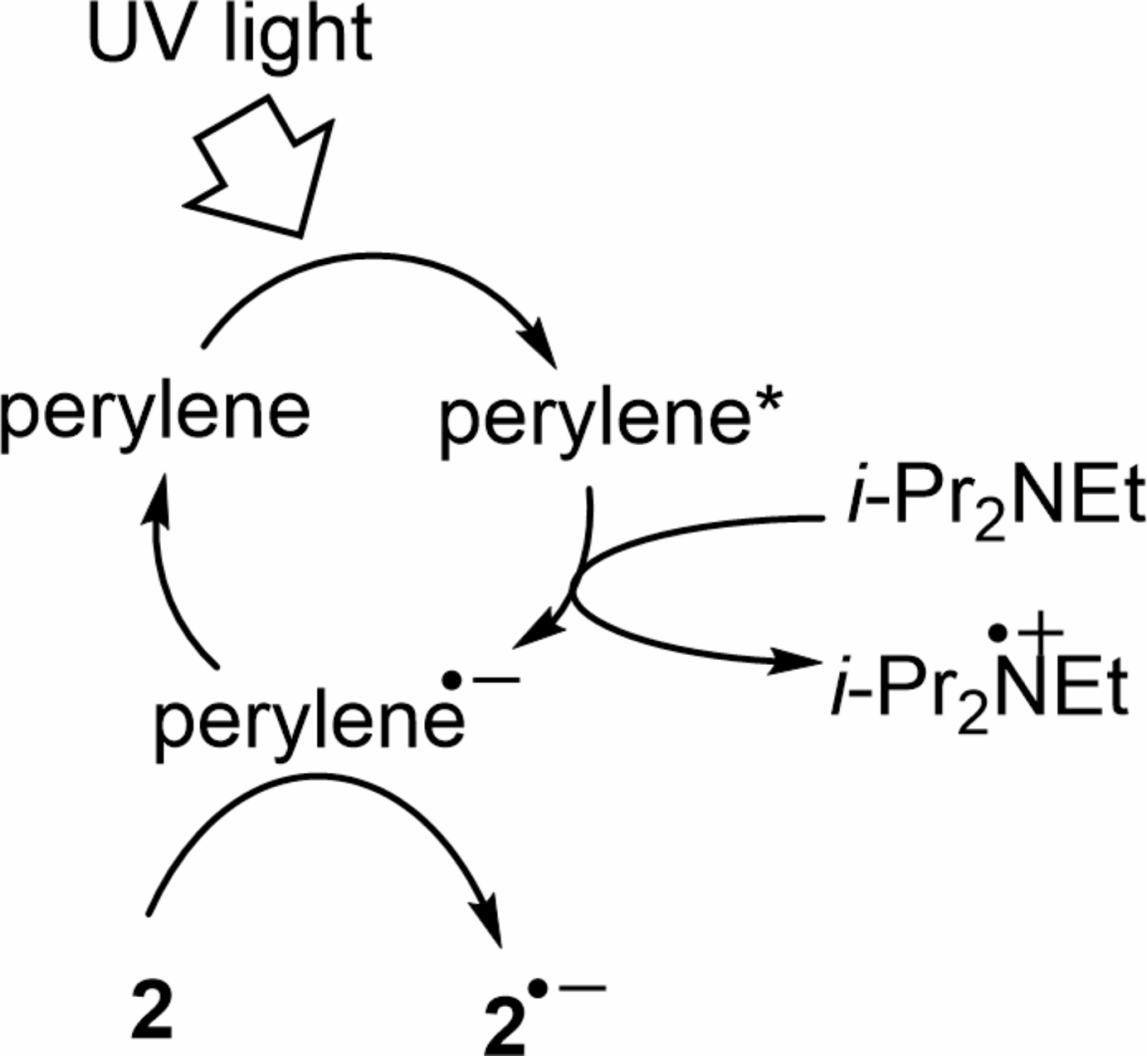
Mechanism of the generation of the anion radical **2**^.−^ by a photoexcited perylene photocatalyst.

The process begins with photoexcitation of the perylene photocatalyst upon UV LED irradiation (Fig. 5 ▸). The excited-state perylene accepts an electron from the sacrificial reductant (*i*-Pr)_2_NEt, generating the anion radical (perylene)^**.**−^. This highly reductive species transfers an electron to **2**, forming the anion radical **2**^**.**−^, which subsequently undergoes transannulation to yield **1**. This occurs *via* consecutive double protonation and one-electron reduction of the inter­mediate anion radical **3**^**.**−^ (Fig. 4 ▸, route 1). Although an alternative pathway involving the formation of the anion radical (**4** + PhSO_2_)^**.**−^*via* S—C bond elongation (route 1′) is also possible, its relatively high activation energy renders it less favourable.

Another proposed pathway involves the radical inter­mediate **5**^**.**^, generated by protonation of **2**^**.**−^. This radical could theoretically lead to **1***via* the inter­mediate **6**^**.**^ through radical transannulation, protonation and single-electron reduction (route 2). However, DFT calculations indicate that rapid elimination of PhSO_2_^**.**^ from **5**^**.**^ is more likely, yielding the elimination product **7**. Similarly, the anion **5**^−^, another potential precursor to **6**^−^, likely undergoes rapid elimination of PhSO_2_^−^, also forming **7**.

## Supplementary Material

Crystal structure: contains datablock(s) I, global. DOI: 10.1107/S205698902500060X/ox2012sup1.cif

Structure factors: contains datablock(s) I. DOI: 10.1107/S205698902500060X/ox2012Isup2.hkl

CCDC reference: 2243325

Additional supporting information:  crystallographic information; 3D view; checkCIF report

## Figures and Tables

**Table 1:** Experimental details

Crystal data	
Chemical formula	C_28_H_22_O_4_S_2_
*M*_r_	486.57
Crystal system, space group	Orthorhombic, *Pna*2_1_
Temperature (K)	293
*a*, *b*, *c* (Å)	17.2598 (3), 10.0898 (1), 13.1810 (2)
*V* (Å^3^)	2295.44 (6)
*Z*	4
Radiation type	Mo *K*α
μ (mm^−1^)	0.27
Crystal size (mm)	0.15 × 0.1 × 0.05

Data collection	
Diffractometer	Rigaku VariMax Saturn724
Absorption correction	Multi-scan (*CrysAlis PRO*; Rigaku OD, 2019 ▸)
*T*_min_, *T*_max_	0.830, 1.000
No. of measured, independent and observed [*I* > 2σ(*I*)] reflections	42446, 7150, 6863
*R*_int_	0.036
(sin θ/λ)_max_ (Å^−1^)	0.736

Refinement	
*R*[*F*^2^ > 2σ(*F*^2^)], *wR*(*F*^2^), *S*	0.031, 0.088, 1.13
No. of reflections	7150
No. of parameters	307
No. of restraints	1
H-atom treatment	H-atom parameters constrained
Δρ_max_, Δρ_min_ (e Å^−3^)	0.46, −0.39
Absolute structure	Flack *x* determined using 3042 quotients [(*I*^+^)−(*I*^−^)]/[(*I*^+^)+(*I*^−^)] (Parsons *et al.*, 2013 ▸)
Absolute structure parameter	−0.048 (15)

Computer programs: *CrysAlis PRO* (Rigaku OD, 2019 ▸), *SHELXT2018* (Sheldrick, 2015*a* ▸), *SHELXL2018* (Sheldrick, 2015*b* ▸) and *OLEX2* (Dolomanov *et al.*, 2009 ▸).

## supplementary crystallographic information

### 8,16-Bis(benzenesulfonyl)tetracyclo[7.7.0.02,7.010,15]hexadeca-2,4,6,10(15),11,13-hexaene Crystal data

**Table:**

C_28_H_22_O_4_S_2_	*D*_x_ = 1.408 Mg m^−^^3^
*M**_r_* = 486.57	Mo *K*α radiation, λ = 0.71073 Å
Orthorhombic, *P**n**a*2_1_	Cell parameters from 35175 reflections
*a* = 17.2598 (3) Å	θ = 2.3–31.6°
*b* = 10.0898 (1) Å	µ = 0.27 mm^−^^1^
*c* = 13.1810 (2) Å	*T* = 293 K
*V* = 2295.44 (6) Å^3^	Plate, yellow
*Z* = 4	0.15 × 0.1 × 0.05 mm
*F*(000) = 1016	

### 8,16-Bis(benzenesulfonyl)tetracyclo[7.7.0.02,7.010,15]hexadeca-2,4,6,10(15),11,13-hexaene Data collection

**Table:**

Rigaku VariMax Saturn724 diffractometer	7150 independent reflections
Radiation source: fine-focus sealed X-ray tube, Enhance (Mo) X-ray Source	6863 reflections with *I* > 2σ(*I*)
Graphite monochromator	*R*_int_ = 0.036
ω scans	θ_max_ = 31.5°, θ_min_ = 2.3°
Absorption correction: multi-scan (CrysAlis PRO; Rigaku OD, 2019)	*h* = −24→25
*T*_min_ = 0.830, *T*_max_ = 1.000	*k* = −14→14
42446 measured reflections	*l* = −18→18

### 8,16-Bis(benzenesulfonyl)tetracyclo[7.7.0.02,7.010,15]hexadeca-2,4,6,10(15),11,13-hexaene Refinement

**Table:**

Refinement on *F*^2^	Hydrogen site location: inferred from neighbouring sites
Least-squares matrix: full	H-atom parameters constrained
*R*[*F*^2^ > 2σ(*F*^2^)] = 0.031	*w* = 1/[σ^2^(*F*_o_^2^) + (0.0578*P*)^2^ + 0.1545*P*] where *P* = (*F*_o_^2^ + 2*F*_c_^2^)/3
*wR*(*F*^2^) = 0.088	(Δ/σ)_max_ = 0.001
*S* = 1.13	Δρ_max_ = 0.46 e Å^−^^3^
7150 reflections	Δρ_min_ = −0.39 e Å^−^^3^
307 parameters	Absolute structure: Flack *x* determined using 3042 quotients [(*I*^+^)-(*I*^-^)]/[(*I*^+^)+(*I*^-^)] (Parsons *et al.*, 2013)
1 restraint	Absolute structure parameter: −0.048 (15)
Primary atom site location: dual	

### 8,16-Bis(benzenesulfonyl)tetracyclo[7.7.0.02,7.010,15]hexadeca-2,4,6,10(15),11,13-hexaene Special details

**Table:**

Geometry. All esds (except the esd in the dihedral angle between two l.s. planes) are estimated using the full covariance matrix. The cell esds are taken into account individually in the estimation of esds in distances, angles and torsion angles; correlations between esds in cell parameters are only used when they are defined by crystal symmetry. An approximate (isotropic) treatment of cell esds is used for estimating esds involving l.s. planes.

### 8,16-Bis(benzenesulfonyl)tetracyclo[7.7.0.02,7.010,15]hexadeca-2,4,6,10(15),11,13-hexaene Fractional atomic coordinates and isotropic or equivalent isotropic displacement parameters (Å^2^)

**Table:**

	*x*	*y*	*z*	*U*_iso_*/*U*_eq_	
S1	0.65385 (3)	0.57943 (4)	0.64924 (4)	0.01544 (10)	
S2	0.63855 (3)	0.13233 (5)	0.35409 (3)	0.01764 (10)	
O1	0.69190 (9)	0.70159 (14)	0.67785 (11)	0.0215 (3)	
O2	0.60389 (9)	0.51442 (16)	0.72176 (12)	0.0228 (3)	
O3	0.60283 (9)	0.21632 (17)	0.27880 (12)	0.0243 (3)	
O4	0.66469 (9)	0.00191 (15)	0.32373 (13)	0.0242 (3)	
C1	0.80081 (11)	0.02918 (18)	0.49055 (17)	0.0204 (4)	
H1	0.810443	−0.015390	0.430098	0.024*	
C2	0.82842 (13)	−0.01963 (19)	0.58237 (19)	0.0236 (4)	
H2	0.857029	−0.097732	0.583286	0.028*	
C3	0.81388 (12)	0.0466 (2)	0.67250 (17)	0.0227 (4)	
H3	0.832998	0.012462	0.733082	0.027*	
C4	0.77070 (11)	0.16425 (19)	0.67352 (15)	0.0187 (4)	
H4	0.760981	0.208656	0.734024	0.022*	
C5	0.74280 (10)	0.21293 (17)	0.58196 (14)	0.0145 (3)	
C6	0.69361 (10)	0.33572 (18)	0.56404 (13)	0.0135 (3)	
H6	0.639491	0.321598	0.583770	0.016*	
C7	0.72795 (10)	0.46170 (18)	0.61359 (14)	0.0134 (3)	
H7	0.757931	0.436692	0.673761	0.016*	
C8	0.78194 (10)	0.51514 (17)	0.53349 (14)	0.0136 (3)	
C9	0.83936 (11)	0.61022 (19)	0.54509 (15)	0.0172 (3)	
H9	0.847046	0.652213	0.607083	0.021*	
C10	0.88527 (12)	0.64112 (19)	0.46118 (16)	0.0205 (4)	
H10	0.923416	0.705802	0.466837	0.025*	
C11	0.87461 (12)	0.57648 (19)	0.36968 (16)	0.0211 (4)	
H11	0.906375	0.597215	0.314948	0.025*	
C12	0.81676 (11)	0.48033 (18)	0.35813 (15)	0.0172 (3)	
H12	0.810090	0.436702	0.296592	0.021*	
C13	0.76950 (10)	0.45154 (17)	0.44072 (13)	0.0131 (3)	
C14	0.70237 (10)	0.35534 (17)	0.44694 (13)	0.0133 (3)	
H14	0.655202	0.391959	0.416477	0.016*	
C15	0.72236 (10)	0.21799 (17)	0.40319 (14)	0.0149 (3)	
H15	0.760873	0.227967	0.349097	0.018*	
C16	0.75839 (10)	0.14641 (18)	0.49152 (15)	0.0156 (3)	
C17	0.60164 (10)	0.60785 (18)	0.53663 (14)	0.0153 (3)	
C18	0.53457 (11)	0.53499 (19)	0.51733 (15)	0.0181 (3)	
H18	0.514311	0.478352	0.566271	0.022*	
C19	0.49848 (11)	0.5487 (2)	0.42331 (17)	0.0203 (4)	
H19	0.454400	0.499324	0.408476	0.024*	
C20	0.52824 (11)	0.63586 (19)	0.35179 (17)	0.0211 (4)	
H20	0.504127	0.644075	0.289013	0.025*	
C21	0.59374 (12)	0.71103 (19)	0.37309 (16)	0.0213 (4)	
H21	0.612296	0.771159	0.325449	0.026*	
C22	0.63150 (11)	0.69642 (18)	0.46544 (15)	0.0180 (3)	
H22	0.676009	0.744952	0.479594	0.022*	
C23	0.57343 (11)	0.11531 (19)	0.45694 (15)	0.0181 (4)	
C24	0.58859 (11)	0.02218 (19)	0.53314 (16)	0.0209 (4)	
H24	0.632003	−0.032214	0.529645	0.025*	
C25	0.53722 (13)	0.0128 (2)	0.61426 (17)	0.0257 (4)	
H25	0.545787	−0.049515	0.665012	0.031*	
C26	0.47331 (13)	0.0959 (2)	0.61982 (19)	0.0279 (5)	
H26	0.439990	0.090482	0.675119	0.033*	
C27	0.45880 (12)	0.1873 (2)	0.5432 (2)	0.0272 (4)	
H27	0.415572	0.242099	0.547105	0.033*	
C28	0.50857 (11)	0.1971 (2)	0.46074 (17)	0.0228 (4)	
H28	0.498734	0.257398	0.408924	0.027*	

### 8,16-Bis(benzenesulfonyl)tetracyclo[7.7.0.02,7.010,15]hexadeca-2,4,6,10(15),11,13-hexaene Atomic displacement parameters (Å^2^)

**Table:**

	*U*^11^	*U*^22^	*U*^33^	*U*^12^	*U*^13^	*U*^23^
S1	0.01851 (18)	0.01647 (19)	0.01135 (19)	0.00111 (15)	0.00189 (16)	−0.00361 (16)
S2	0.01760 (18)	0.0206 (2)	0.0147 (2)	−0.00383 (15)	0.00205 (17)	−0.00729 (18)
O1	0.0270 (7)	0.0187 (6)	0.0190 (7)	0.0009 (5)	−0.0016 (6)	−0.0079 (5)
O2	0.0245 (7)	0.0286 (7)	0.0152 (7)	0.0023 (6)	0.0086 (5)	0.0005 (6)
O3	0.0242 (7)	0.0331 (8)	0.0156 (7)	−0.0028 (6)	−0.0030 (5)	−0.0040 (6)
O4	0.0249 (7)	0.0230 (7)	0.0248 (8)	−0.0052 (6)	0.0073 (6)	−0.0135 (6)
C1	0.0180 (8)	0.0162 (8)	0.0270 (10)	−0.0014 (6)	0.0045 (7)	−0.0050 (7)
C2	0.0224 (9)	0.0150 (8)	0.0334 (11)	0.0013 (7)	0.0025 (8)	0.0015 (8)
C3	0.0241 (9)	0.0195 (8)	0.0245 (10)	−0.0002 (7)	−0.0006 (8)	0.0059 (7)
C4	0.0229 (8)	0.0173 (8)	0.0159 (9)	−0.0018 (7)	0.0019 (7)	0.0029 (6)
C5	0.0154 (7)	0.0134 (7)	0.0148 (8)	−0.0015 (6)	0.0020 (6)	0.0002 (6)
C6	0.0147 (7)	0.0146 (7)	0.0112 (8)	−0.0007 (6)	0.0015 (6)	−0.0016 (6)
C7	0.0153 (7)	0.0143 (7)	0.0105 (7)	−0.0005 (6)	0.0002 (6)	−0.0018 (6)
C8	0.0150 (7)	0.0132 (7)	0.0125 (8)	0.0009 (6)	0.0012 (6)	0.0000 (6)
C9	0.0207 (8)	0.0146 (7)	0.0163 (8)	−0.0025 (6)	0.0002 (7)	−0.0017 (7)
C10	0.0226 (8)	0.0168 (8)	0.0220 (9)	−0.0062 (7)	0.0030 (7)	0.0011 (7)
C11	0.0236 (9)	0.0220 (9)	0.0177 (9)	−0.0036 (7)	0.0056 (7)	0.0033 (7)
C12	0.0218 (8)	0.0183 (7)	0.0114 (7)	−0.0007 (6)	0.0018 (7)	0.0012 (7)
C13	0.0149 (7)	0.0129 (7)	0.0115 (8)	0.0002 (6)	0.0005 (6)	−0.0006 (6)
C14	0.0147 (7)	0.0147 (7)	0.0106 (7)	−0.0006 (6)	0.0000 (6)	−0.0028 (6)
C15	0.0157 (7)	0.0162 (8)	0.0128 (8)	−0.0027 (6)	0.0020 (6)	−0.0042 (6)
C16	0.0140 (7)	0.0149 (7)	0.0179 (9)	−0.0018 (6)	0.0017 (6)	−0.0023 (6)
C17	0.0158 (7)	0.0152 (7)	0.0150 (8)	0.0023 (6)	0.0000 (6)	−0.0029 (6)
C18	0.0167 (8)	0.0180 (8)	0.0194 (9)	−0.0002 (6)	0.0036 (7)	−0.0009 (7)
C19	0.0166 (8)	0.0200 (8)	0.0243 (10)	0.0013 (7)	−0.0022 (7)	−0.0030 (7)
C20	0.0211 (8)	0.0229 (9)	0.0193 (9)	0.0056 (7)	−0.0028 (8)	−0.0006 (7)
C21	0.0249 (9)	0.0188 (8)	0.0202 (9)	0.0010 (7)	0.0012 (7)	0.0034 (7)
C22	0.0189 (8)	0.0138 (7)	0.0212 (9)	0.0004 (6)	0.0011 (7)	−0.0008 (7)
C23	0.0164 (8)	0.0199 (8)	0.0181 (9)	−0.0052 (6)	0.0037 (7)	−0.0080 (7)
C24	0.0198 (8)	0.0201 (8)	0.0227 (10)	−0.0032 (7)	0.0031 (7)	−0.0071 (7)
C25	0.0282 (10)	0.0283 (10)	0.0205 (9)	−0.0095 (8)	0.0044 (8)	−0.0046 (8)
C26	0.0241 (9)	0.0334 (11)	0.0262 (10)	−0.0104 (8)	0.0101 (8)	−0.0128 (9)
C27	0.0187 (8)	0.0280 (10)	0.0348 (11)	−0.0027 (7)	0.0058 (8)	−0.0116 (9)
C28	0.0180 (8)	0.0216 (8)	0.0288 (11)	−0.0032 (7)	0.0007 (8)	−0.0065 (8)

### 8,16-Bis(benzenesulfonyl)tetracyclo[7.7.0.02,7.010,15]hexadeca-2,4,6,10(15),11,13-hexaene Geometric parameters (Å, º)

**Table:**

S1—O1	1.4467 (15)	C11—C12	1.400 (3)
S1—O2	1.4448 (15)	C12—H12	0.9300
S1—C7	1.8077 (18)	C12—C13	1.391 (2)
S1—C17	1.7600 (19)	C13—C14	1.514 (2)
S2—O3	1.4433 (17)	C14—H14	0.9800
S2—O4	1.4475 (15)	C14—C15	1.540 (2)
S2—C15	1.8051 (18)	C15—H15	0.9800
S2—C23	1.7693 (19)	C15—C16	1.505 (3)
C1—H1	0.9300	C17—C18	1.395 (3)
C1—C2	1.391 (3)	C17—C22	1.394 (3)
C1—C16	1.391 (3)	C18—H18	0.9300
C2—H2	0.9300	C18—C19	1.394 (3)
C2—C3	1.386 (3)	C19—H19	0.9300
C3—H3	0.9300	C19—C20	1.388 (3)
C3—C4	1.402 (3)	C20—H20	0.9300
C4—H4	0.9300	C20—C21	1.390 (3)
C4—C5	1.389 (3)	C21—H21	0.9300
C5—C6	1.520 (2)	C21—C22	1.388 (3)
C5—C16	1.394 (2)	C22—H22	0.9300
C6—H6	0.9800	C23—C24	1.400 (3)
C6—C7	1.547 (2)	C23—C28	1.392 (3)
C6—C14	1.563 (2)	C24—H24	0.9300
C7—H7	0.9800	C24—C25	1.392 (3)
C7—C8	1.508 (3)	C25—H25	0.9300
C8—C9	1.388 (2)	C25—C26	1.388 (3)
C8—C13	1.398 (2)	C26—H26	0.9300
C9—H9	0.9300	C26—C27	1.391 (4)
C9—C10	1.396 (3)	C27—H27	0.9300
C10—H10	0.9300	C27—C28	1.388 (3)
C10—C11	1.383 (3)	C28—H28	0.9300
C11—H11	0.9300		
			
O1—S1—C7	107.84 (9)	C8—C13—C14	111.38 (15)
O1—S1—C17	108.27 (9)	C12—C13—C8	119.92 (16)
O2—S1—O1	119.03 (9)	C12—C13—C14	128.70 (16)
O2—S1—C7	107.21 (9)	C6—C14—H14	111.8
O2—S1—C17	109.05 (9)	C13—C14—C6	102.05 (14)
C17—S1—C7	104.48 (9)	C13—C14—H14	111.8
O3—S2—O4	118.48 (10)	C13—C14—C15	112.65 (14)
O3—S2—C15	107.92 (9)	C15—C14—C6	106.10 (14)
O3—S2—C23	108.21 (10)	C15—C14—H14	111.8
O4—S2—C15	106.53 (9)	S2—C15—H15	109.4
O4—S2—C23	108.76 (9)	C14—C15—S2	112.68 (12)
C23—S2—C15	106.31 (9)	C14—C15—H15	109.4
C2—C1—H1	120.9	C16—C15—S2	112.26 (12)
C2—C1—C16	118.26 (19)	C16—C15—C14	103.58 (14)
C16—C1—H1	120.9	C16—C15—H15	109.4
C1—C2—H2	119.6	C1—C16—C5	121.24 (18)
C3—C2—C1	120.88 (18)	C1—C16—C15	128.22 (18)
C3—C2—H2	119.6	C5—C16—C15	110.53 (15)
C2—C3—H3	119.6	C18—C17—S1	119.53 (15)
C2—C3—C4	120.8 (2)	C22—C17—S1	118.87 (14)
C4—C3—H3	119.6	C22—C17—C18	121.45 (18)
C3—C4—H4	120.8	C17—C18—H18	120.6
C5—C4—C3	118.38 (18)	C19—C18—C17	118.74 (18)
C5—C4—H4	120.8	C19—C18—H18	120.6
C4—C5—C6	128.07 (17)	C18—C19—H19	120.0
C4—C5—C16	120.38 (17)	C20—C19—C18	120.08 (18)
C16—C5—C6	111.55 (16)	C20—C19—H19	120.0
C5—C6—H6	111.9	C19—C20—H20	119.7
C5—C6—C7	112.95 (14)	C19—C20—C21	120.6 (2)
C5—C6—C14	101.69 (14)	C21—C20—H20	119.7
C7—C6—H6	111.9	C20—C21—H21	120.0
C7—C6—C14	106.00 (14)	C22—C21—C20	120.05 (19)
C14—C6—H6	111.9	C22—C21—H21	120.0
S1—C7—H7	109.4	C17—C22—H22	120.5
C6—C7—S1	112.24 (12)	C21—C22—C17	118.98 (17)
C6—C7—H7	109.4	C21—C22—H22	120.5
C8—C7—S1	112.60 (12)	C24—C23—S2	119.74 (15)
C8—C7—C6	103.59 (14)	C28—C23—S2	118.75 (16)
C8—C7—H7	109.4	C28—C23—C24	121.51 (19)
C9—C8—C7	127.69 (17)	C23—C24—H24	120.7
C9—C8—C13	121.56 (17)	C25—C24—C23	118.52 (19)
C13—C8—C7	110.70 (15)	C25—C24—H24	120.7
C8—C9—H9	120.9	C24—C25—H25	119.8
C8—C9—C10	118.20 (18)	C26—C25—C24	120.4 (2)
C10—C9—H9	120.9	C26—C25—H25	119.8
C9—C10—H10	119.7	C25—C26—H26	119.8
C11—C10—C9	120.66 (18)	C25—C26—C27	120.4 (2)
C11—C10—H10	119.7	C27—C26—H26	119.8
C10—C11—H11	119.5	C26—C27—H27	119.9
C10—C11—C12	121.08 (19)	C28—C27—C26	120.3 (2)
C12—C11—H11	119.5	C28—C27—H27	119.9
C11—C12—H12	120.7	C23—C28—H28	120.5
C13—C12—C11	118.53 (18)	C27—C28—C23	118.9 (2)
C13—C12—H12	120.7	C27—C28—H28	120.5
			
S1—C7—C8—C9	73.1 (2)	C7—S1—C17—C22	−80.77 (16)
S1—C7—C8—C13	−109.25 (15)	C7—C6—C14—C13	24.61 (17)
S1—C17—C18—C19	−173.37 (14)	C7—C6—C14—C15	142.74 (13)
S1—C17—C22—C21	174.87 (15)	C7—C8—C9—C10	177.19 (18)
S2—C15—C16—C1	72.1 (2)	C7—C8—C13—C12	−175.93 (16)
S2—C15—C16—C5	−106.73 (15)	C7—C8—C13—C14	3.8 (2)
S2—C23—C24—C25	178.68 (15)	C8—C9—C10—C11	−1.3 (3)
S2—C23—C28—C27	−177.74 (15)	C8—C13—C14—C6	−17.84 (18)
O1—S1—C7—C6	−170.12 (12)	C8—C13—C14—C15	−131.19 (16)
O1—S1—C7—C8	−53.69 (15)	C9—C8—C13—C12	1.9 (3)
O1—S1—C17—C18	−150.48 (15)	C9—C8—C13—C14	−178.38 (16)
O1—S1—C17—C22	33.97 (17)	C9—C10—C11—C12	1.2 (3)
O2—S1—C7—C6	60.58 (15)	C10—C11—C12—C13	0.5 (3)
O2—S1—C7—C8	177.01 (13)	C11—C12—C13—C8	−2.0 (3)
O2—S1—C17—C18	−19.58 (17)	C11—C12—C13—C14	178.35 (18)
O2—S1—C17—C22	164.87 (14)	C12—C13—C14—C6	161.85 (18)
O3—S2—C15—C14	56.45 (15)	C12—C13—C14—C15	48.5 (2)
O3—S2—C15—C16	172.94 (12)	C13—C8—C9—C10	−0.2 (3)
O3—S2—C23—C24	169.95 (15)	C13—C14—C15—S2	−152.10 (13)
O3—S2—C23—C28	−11.08 (18)	C13—C14—C15—C16	86.35 (17)
O4—S2—C15—C14	−175.33 (13)	C14—C6—C7—S1	98.89 (14)
O4—S2—C15—C16	−58.84 (15)	C14—C6—C7—C8	−22.85 (17)
O4—S2—C23—C24	40.02 (18)	C14—C15—C16—C1	−166.03 (18)
O4—S2—C23—C28	−141.01 (15)	C14—C15—C16—C5	15.10 (19)
C1—C2—C3—C4	0.2 (3)	C15—S2—C23—C24	−74.34 (17)
C2—C1—C16—C5	−0.9 (3)	C15—S2—C23—C28	104.63 (16)
C2—C1—C16—C15	−179.62 (18)	C16—C1—C2—C3	0.2 (3)
C2—C3—C4—C5	0.0 (3)	C16—C5—C6—C7	−129.03 (16)
C3—C4—C5—C6	178.56 (17)	C16—C5—C6—C14	−15.86 (18)
C3—C4—C5—C16	−0.7 (3)	C17—S1—C7—C6	−55.08 (14)
C4—C5—C6—C7	51.7 (2)	C17—S1—C7—C8	61.35 (14)
C4—C5—C6—C14	164.83 (18)	C17—C18—C19—C20	−1.5 (3)
C4—C5—C16—C1	1.1 (3)	C18—C17—C22—C21	−0.6 (3)
C4—C5—C16—C15	−179.91 (16)	C18—C19—C20—C21	−0.5 (3)
C5—C6—C7—S1	−150.61 (13)	C19—C20—C21—C22	2.0 (3)
C5—C6—C7—C8	87.66 (17)	C20—C21—C22—C17	−1.4 (3)
C5—C6—C14—C13	−93.66 (15)	C22—C17—C18—C19	2.1 (3)
C5—C6—C14—C15	24.47 (17)	C23—S2—C15—C14	−59.45 (15)
C6—C5—C16—C1	−178.24 (16)	C23—S2—C15—C16	57.04 (15)
C6—C5—C16—C15	0.7 (2)	C23—C24—C25—C26	−1.1 (3)
C6—C7—C8—C9	−165.41 (17)	C24—C23—C28—C27	1.2 (3)
C6—C7—C8—C13	12.25 (19)	C24—C25—C26—C27	1.6 (3)
C6—C14—C15—S2	97.06 (15)	C25—C26—C27—C28	−0.6 (3)
C6—C14—C15—C16	−24.50 (17)	C26—C27—C28—C23	−0.8 (3)
C7—S1—C17—C18	94.78 (16)	C28—C23—C24—C25	−0.3 (3)
